# Plant Regeneration and Cellular Behaviour Studies in *Celosia cristata* Grown *In Vivo* and *In Vitro*

**DOI:** 10.1100/2012/359413

**Published:** 2012-04-19

**Authors:** Rosna Mat Taha, Sharifah Nurashikin Wafa

**Affiliations:** Institute of Biological Sciences, Faculty of Science, University of Malaya, 50603 Kuala Lumpur, Malaysia

## Abstract

Tissue culture studies of *Celosia cristata* were established from various explants and the effects of various hormones on morphogenesis of this species were examined. It was found that complete plant regeneration occurred at highest percentage on MS medium supplemented with 2.0 mg/L NAA and 1.5 mg/L BAP, with the best response showed by shoot explants. *In vitro* flowering was observed on MS basal medium after six weeks. The occurrence of somaclonal variation and changes in cellular behavior from *in vivo* and *in vitro* grown plants were investigated through cytological studies and image analysis. It was observed that Mitotic Index (MI), mean chromosome numbers, and mean nuclear to cell area ratio of *in vitro* root meristem cells were slightly higher compared to *in vivo* values. However, *in vitro* plants produced lower mean cell areas but higher nuclear areas when compared to *in vivo* plants. Thus, no occurrence of somaclonal variation was detected, and this was supported by morphological features of the *in vitro* plants.

## 1. Introduction

*Celosia cristata* is native to South America and now is widespread in Asia, especially, Malaysia. It is now becoming more important as ornamental plant and often used for landscaping and roadside plants due to the various attractive colours of the flowers. Due to its high demands, *in vitro* cloning has been introduced for this species. *In vitro* cloning has many advantages. Specifically, propagation *in vitro* is more rapid than *in vivo*, with the possibility of producing disease-free plants using little starting materials. In addition, through the use of *in vitro* methods, the effect of the seasons can be eliminated and year-round production can be achieved, or a new cultivar can be made commercially available more quickly [1]. *Celosia cristata* was found to be able to produce purplish or reddish pigment in tissue culture system and when analysed was found to contain cyanidin, a kind of anthocyanin. This species was also capable of producing *in vitro* flowers after only 6 weeks in culture [2]. Because of these abilities, and the popularity of this plant, *Celosia cristata *was cultured to study the morphogenesis *in vitro* and the effect of various hormones on plant regeneration in attempt to achieve efficient mass propagation of this species. Cellular behavior studies, such as mean cell and nuclear areas, Mitotic index (MI) and ploidy level are also very important in plants, for example, for differentiating between embryogenic and nonembryogenic calli [3]. In addition, the rate of cell division correlates with growth rates [4]. Some cellular investigations have revealed the occurrence of somaclonal variation, even at an early stage. Hence, in the current work, cellular behaviours, such as Mitotic index (MI), chromosome counts, mean cell, and nuclear areas and their ratios were also investigated, in regenerants and intact plants, to determine if somaclonal variations occurred during the culture protocols. Although *in vitro* flowering of this species has been previously reported [2], changes in cellular behaviour during *in vitro* flowering have never been reported before. Therefore, the objectives of the present work are to establish efficient regeneration system for this species, to compare cellular behaviours in root meristem cells of *in vivo* and *in vitro *grown plants, to detect whether any somaclonal variation had occurred during tissue culture protocols and finally, to observe the changes in cellular behavior during *in vitro* flowering.

## 2. Materials and Methods

### 2.1. The Effects of Various Hormones on Tissue Culture of *Celosia cristata*

*Celosia cristata *seeds were obtained from nurseries around Kuala Lumpur and were sterilised in standard tissue culture protocols [5, 6]. Seeds were treated with 100%, 70%, 50%, 20%, and 10% (v/v) commercial bleach (chlorox) for 3-4 min at each concentration. At 100% (v/v) concentrations of chlorox, 3 drops of Tween-20 were added, in order to facilitate sterilisation. Surface sterilisation of the seeds was accomplished by submersion in 70% (v/v) ethanol, followed by rinsing 3 times with sterile distilled water under aseptic conditions using a laminar flow chamber. Subsequently, seeds were cultured on MS basal medium. One-month-old aseptic seedlings were used as explant sources. Leaves (0.5 × 0.5 cm), stems (0.5 cm), root segments (0.5 cm), and shoot tips (1.0 cm) were excised from aseptic seedlings to initiate cultures. MS medium supplemented with various hormones, such as benzyl aminopurine (BAP), naphthalene acetic acid (NAA), indole acetic acid (IAA), kinetin and zeatin were used at different concentrations with different combinations. Throughout the experiment, the culture media used was Murashige and Skoog (MS) media supplemented with 3% sucrose and 0.8% agar. The pH of the media was adjusted to 5.8 ± 0.1 before autoclaving at 121°C for 15 min. Twenty replicates were used for each experiment. Cultures were maintained at 25 ± 1°C with 16 h light and 8 h dark, except for *in vitro* flowering experiments, where cultures were subjected to 12 h light and 12 h dark. Regenerants were acclimatised in garden soil as previously described [7]. Full-grown plantlets were transferred to flower pots containing a mixture of black soil and sand at a ratio of 3 : 1 and covered with transparent polyethylene bags to prevent plant dehydration. Plantlets were kept in the culture room at 25 ± 1°C under 16-hour light and 8-hour dark. After 2 weeks, well-grown and healthy plantlets were transferred to a greenhouse.

### 2.2. Cytological Investigations on Roots Grown *In Vivo* versus *In Vitro*

One hundred (100) seeds of *Celosia cristata* were germinated on moist cotton wool in Petri dishes for 2 weeks, and also on MS basal medium (for comparison), to obtain standard growth curves for the primary roots. The primary root length of the population was measured once per day at a fixed time and the mean root length of each sample was recorded and was plotted against time. Roots were then cut and soaked in alcohol and acetic acid (3 : 1) overnight for preservation. Based on the standard curve, the mean root length of the sample displaying the highest growth rate before the emergence of secondary roots was determined. For subsequent experiments, the mean root length of the sample was chosen, fixed, and made into permanent slides. Permanent slides were prepared by soaking primary roots in 5 M Hydrochloric acid (HCl) and stained with Feulgen. Subsequently, roots were transferred onto slides, mounted with 45% (v/v) acetic acid, sprayed with freeze spray, and rinsed. Cover slides were then mounted on the slides by DPX (Di-N-Butyle Phthalate in Xylene). Primary roots produced *in vitro* were also made into permanent slides and analysed for cellular behaviours, such as Mitotic index, chromosome count, mean nuclear and cell areas, and their ratios. For measurement of mean cell and nuclear areas, standard protocols using an image analyser were followed. The experiments were conducted using a light microscope (Zeiss Axioscope, Germany) connected to a Sony video camera, images from 500 interphase cells were captured and transferred to a host computer for image analyzing. The system was supported by two macros programs, that are, DNA for calculation of DNA C value and VIDAS for cell and nuclear area measurement (Kontron Electronic, Germany). The DNA C value was determined by calibrating the integrated optical density with reference nuclei [3].

## 3. Results and Discussion

**Figure 1:**
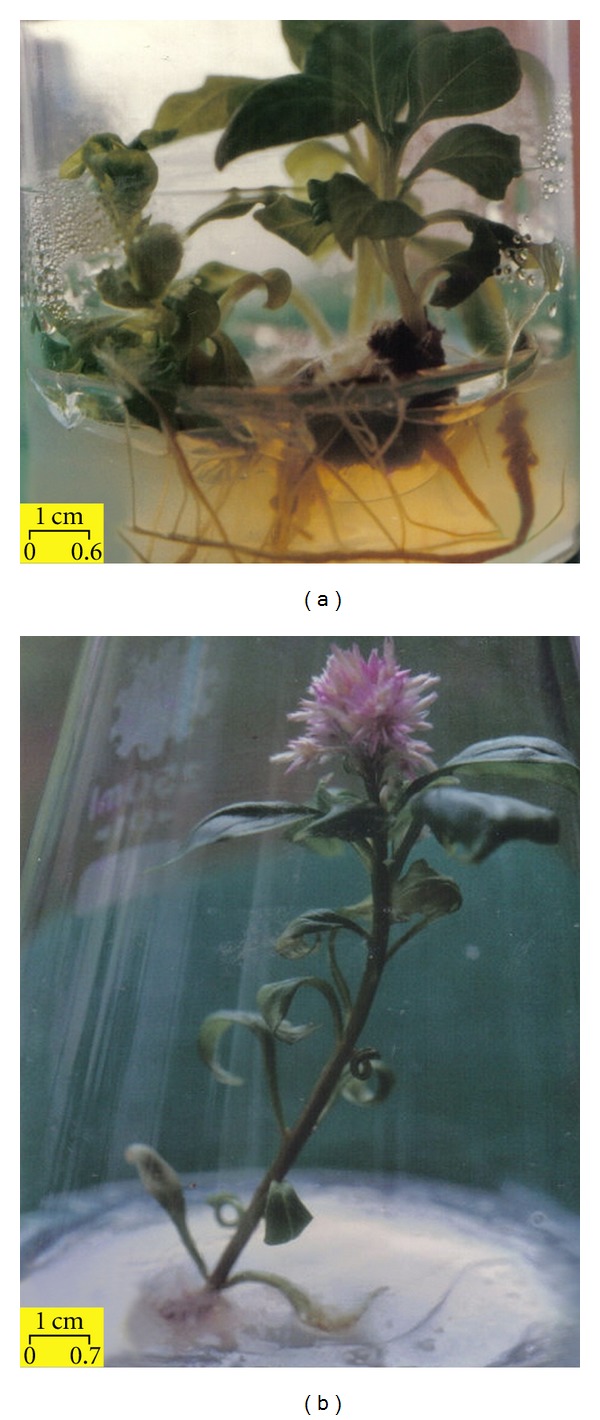
(a) Complete *in vitro* plant regeneration of *Celosia cristata* derived from a shoot explant cultured in MS basal medium after 35 days of culture. (b) *In vitro* flowering of *Celosia cristata* in MS basal medium after 2 months of culture at 25 ± 1°C under 12 h light and 12 h dark.

In general, callus growth in *Celosia cristata* was observed after as early as 7–10 days in culture. Stem, leaf, shoot, and even root explants were able to produce reddish purple calli on MS medium containing 0.5–2.0 mg/L BAP and 0.5 mg/L NAA. When shoot explants were cultured on MS basal medium (Figure 1(a)) and MS medium supplemented with 2.0 mg/L NAA, 1.0 mg/L NAA + 0.5 mg/L BAP, or 1.5 mg/L NAA + 2.0 mg/L BAP, or 1.0 mg/L IAA, 1.0 mg/L Kinetin + 1.0 mg/L Zeatin; 100% direct regeneration of *C. cristata* was obtained in 35 days. In addition, direct regeneration of *C. cristata *was achieved in 20 days when shoot explants were cultured on MS medium supplemented with 2.0 mg/L NAA + 0.5 mg/L BAP, or 2.0 mg/L NAA + 1.5 mg/L BAP. Roots were obtained on most of the media tested; for example, MS supplemented with 0.5–2.0 mg/L NAA and 0.5–2.0 mg/L BAP, or MS basal medium. *In vitro* flowering (Figure 1(b)) was observed when shoot explants were cultured on MS basal media after being exposed to 12 h light and 12 h dark (Data not shown). Some plantlets with abnormal stems (swollen) were observed when shoot tips were cultured on MS supplemented with 0.5–2.0 mg/L BAP, applied singly. The responses of various explants to different concentrations of hormones are shown in Table 1.

**Figure 2:**
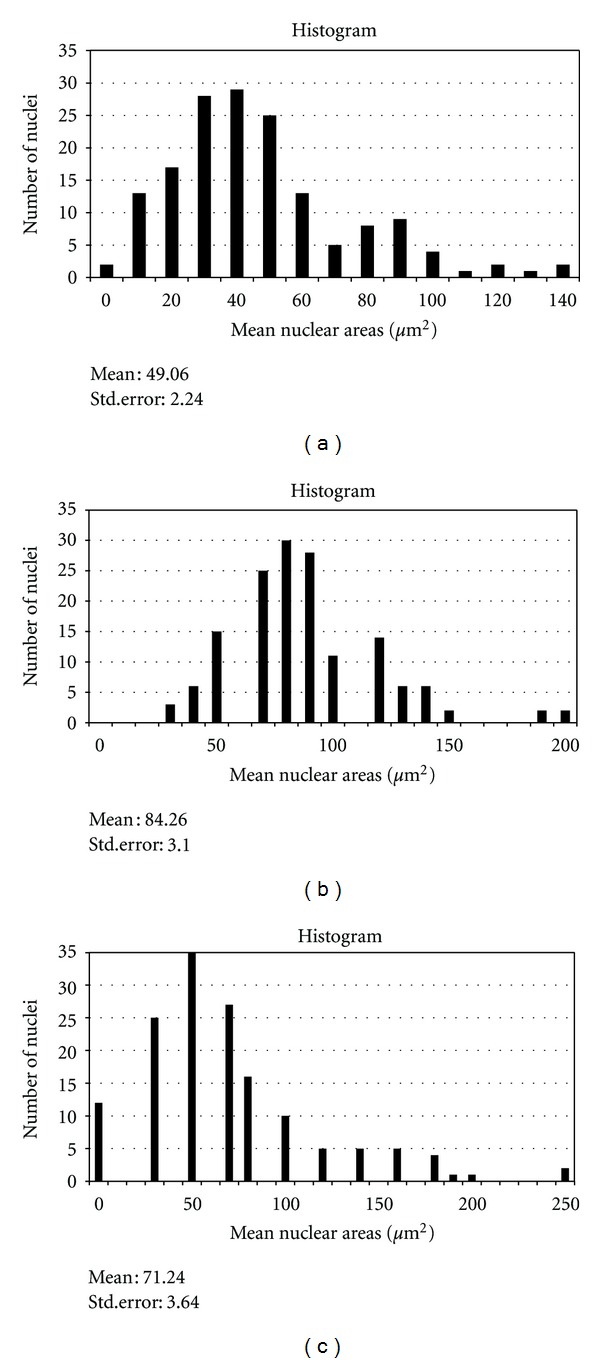
(a) Mean nuclear areas for *in vivo* primary root cells. (b) Mean nuclear areas for *in vitro *primary root cells (normal). (c) Mean nuclear areas for *in vitro* primary root cells (abnormal).

**Figure 3:**
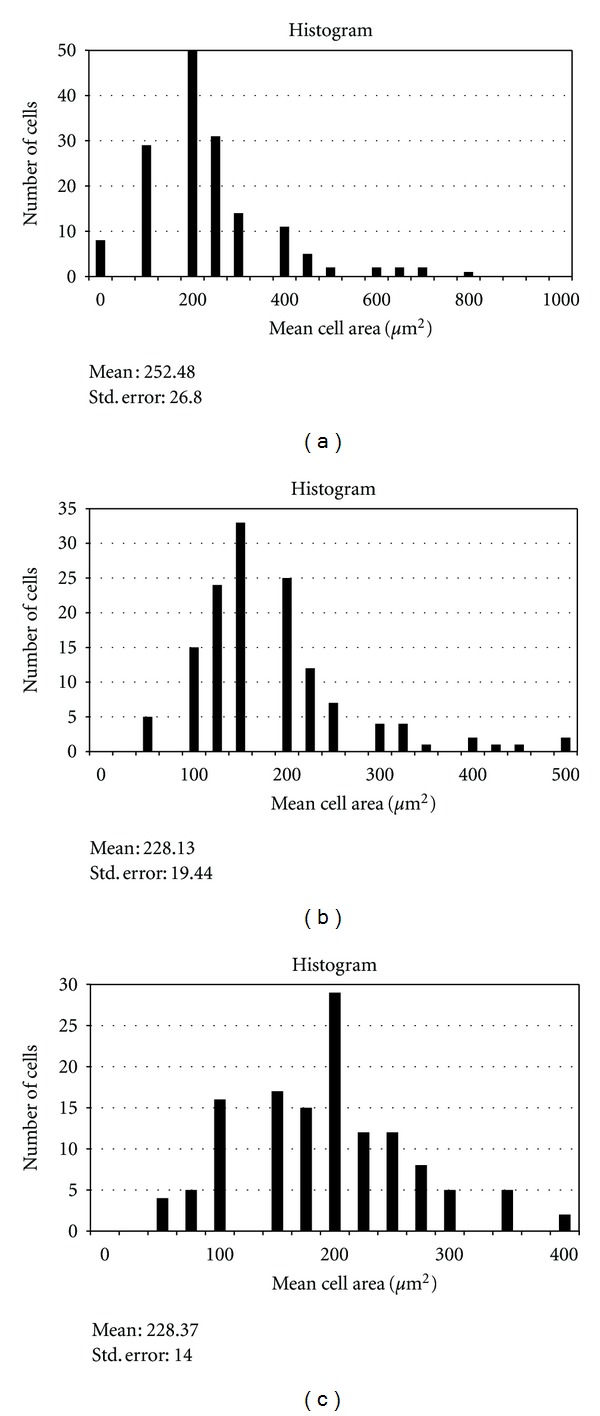
(a) Mean cell areas for *in vivo* primary root cells. (b) Mean cell areas for *in vitro* primary root cells (normal). (c) Mean cell areas for *in vitro* primary root cells (abnormal).

The mean primary root length of the sample after 7 days of germination was determined. In subsequent experiments, only primary roots which achieved this mean root length were chosen. Permanent slides of the primary roots were prepared as previously described [8]. In meristematic cells of intact roots which achieved the mean root length of the sample, the mean MI value was determined to be 39.03 ± 4.67. The chromosome count was 33.23 (Table 2) and the mean cell and nuclear areas were 252.48 ± 25.88 *μ*m^2^ and 49.06 ± 2.16 *μ*m^2^, respectively (Table 3; Figures 2(a) and 3(a)). Primary roots obtained *in vitro* after 4 weeks were made into squashed preparations as previously described [9]. The MI value for normal *in vitro* grown plantlets was 45.64 ± 3.16 and the chromosome count was 36.05 (Table 2), in agreement with the chromosome number of intact *C. cristata* plants reported by Grant (1954) [10]. The mean cell and nuclear areas for root meristem cells from *in vitro* grown plants were 228.13 ± 19.44 *μ*m^2^ and 84.26 ± 3.07 *μ*m^2^, respectively, while the ratio of mean nuclear to cell area was 0.37 ± 0.01 (Figures 2(b) and 3(b); Table 3). In abnormal plantlets (swollen stems), the MI was found to be 43.65 ± 2.75, with a chromosome count of 36.98 (Table 2). Plantlets that exhibited *in vitro* flowering had a mean MI value of 46.35 ± 0.99, with a chromosome count of 42.07, higher than the value of 33.23 found for *in vivo* grown plants. In contrast, nonflowering regenerants had a slightly lower mean MI value (42.27 ± 2.86) and a lower mean chromosome number (38.24) compared to *in vitro* flowering plants (Table 2). Because the *in vivo* mean chromosome count was 36, some changes may have taken place during tissue culture with respect to cellular and nuclear behaviour: perhaps due to media, hormones, environmental cultural factors, and/or explants, and so forth. Consistent with this notion, in a study by Tabur and Oney [11], high concentrations of artificial fertilisers had negative effects on the Mitotic index and chromosome behaviour in *Vicia hybrida* L. 

Figures 2(c) and 3(c) show the mean nuclear and cell areas for root cells from abnormal plants. While the mean nuclear area was observed to be increased (71.24 ± 3.56) versus intact plants, the mean cell area decreased and was almost equal to that observed for *in vitro* cells. Although the ratio of mean nuclear to cell area was larger than that found for intact plants, this ratio was slightly smaller than that measured for *in vitro* plants (Table 3). These results suggest that cell and nuclear areas may have changed independently of each other: since mean cell areas decreased while mean nuclear areas increased. This is in agreement with the work of Taha and Francis [12] on *Vicia faba. *Furthermore the results obtained in the present study is supported by the findings of Thomas and Davidson [13], whereby they found that the relationship between nuclear and cell size of *Vicia faba* can change abruptly without affecting mitotic activity, and the nuclei can remain constant in size, despite a decrease in cell sizes. In addition, Taha and Francis also reported that chromosome numbers and MI values increased with culture age, consistent with results observed in the present study. This could be due to cellular instability upon transfer from one environment to another, in this case from *in vivo* to *in vitro* conditions. Besides, the cells could experience shock effect during transfer. Possibly the mechanisms regulating cell and nuclear size and other cellular parameters are disturbed when cells are brought into culture, coupled with a loss of the organising influences imposed on cells by intact plants. 

 MI values observed in the present study for *C. cristata *were very high compared to many other species: for example in *V. faba, *the MI value was only 25% [14]. This is consistent with the readiness of *C. cristata* to respond actively in culture, as shown in Table 1, whereas in *V. Faba*, regeneration was not easily achieved. All *C. cristata* explant sources could regenerate or were able to produce shoots *in vitro*, whereas, in *V. faba*, it is comparatively more difficult to induce callus and regeneration *in vitro *[15]. Unfortunately, very few reports have been published in the literature discussing changes to cellular behaviour when cells are grown in tissue culture systems. The cellular behaviour parameters investigated in the present study do not appear to follow any particular pattern. Even in abnormal stems, the MI, mean chromosome number, mean cell and nuclear areas, and their ratio were nearly similar to the normal and *in vitro* flowering plantlets. Interestingly, nearly all cellular parameters examined increased *in vitro*, with the exception of mean cell area. However, this decrease in cell area did not hinder callus formation, plant regeneration, or *in vitro* flowering in *C. cristata*. Usually, cultures that form shoots or roots exhibit reduced cell sizes for example in shoot forming tobacco callus cultures [16]. It is hoped that more research on the cellular behaviour of plant species will be carried out, to pinpoint exactly the pattern of changes that occur when cells are transferred from *in vivo* to *in vitro* environments. Cellular behaviour is also important for the regeneration potential of a particular species. 

## 4. Conclusion

Direct regeneration and *in vitro* flowering of *C. cristata* have been obtained from shoot explants cultured on MS basal medium. The highest percentage of plant regeneration was achieved on MS medium supplemented with 2.0 mg/L NAA and 1.5 mg/L BAP. Use of BAP alone on MS medium was associated with abnormalities during plant regeneration. No differences were observed with respect to mitotic index (MI), chromosome number, mean nuclear, and cell areas between normal and abnormal plants. Further investigations must be conducted to enable a better understanding of cellular behaviour in relation to plant regeneration and *in vitro* flowering. 

## Figures and Tables

**Table 1:** Effects of different hormones on the tissue culture of *Celosia cristata* initiated from various explants maintained at 25 ± 1°C with 16 h light and 8 h dark.

Hormone concentration (mg/l)					Explants	Callus (%)	Direct regeneration (%)	Observations
NAA	BAP	IAA	KIN	ZEA				
—	—	—	—	—	Leaves	—	—	—
					Stem	—	—	—
					Root	—	—	—
					Shoot	—	100	Multiple shoots and roots after 35 days

—	0.5	—	—	—	Leaves	25	—	White callus and root
					Stem	50	—	Orange callus
					Root	—	—	—
					Shoot	—	90	Multiple shoots, abnormal stem

—	1.0	—	—	—	Leaves	65	—	White callus and later dormant
					Stem	100	—	Red, black, and white callus
					Root	—	—	—
					Shoot	—	80	multiple shoots, abnormal stem

—	1.5	—	—	—	Leaves	65	—	White callus and later dormant
					Stem	100	—	White, green, and black callus
					Root	50	—	Callus
					Shoot	—	100	Multiple shoots, abnormal stem

—	2.0	—	—	—	Leaves	40	—	White callus and later dormant
					Stem	100	10	Orange callus and shoot, necrosis
					Root	65	—	Callus
					Shoot	—	100	Multiple shoots, abnormal stem

0.5	—	—	—	—	Leaves	95	—	Yellow callus and root
					Stem	100	—	White callus, necrosis
					Root	55	—	Callus
					Shoot	—	100	Multiple shoots

1.0	—	—	—	—	Leaves	20	—	White callus and roots
					Stem	30	—	Callus and roots
					Root	—	—	Roots
					Shoot	—	85	Direct regeneration after 35 days

1.5	—	—	—	—	Leaves	10	—	Callus and roots
					Stem	80	—	Callus and roots
					Root	30	—	Roots
					Shoot	—	50	Multiple shoots and roots

2.0	—	—	—	—	Leaves	5	—	Callus and roots
					Stem	15	—	Callus and roots
					Root	5	—	Roots
					Shoot	—	100	Direct regeneration after 35 days

0.5	0.5	—	—	—	Leaves	40	—	White callus and roots
					Stem	100	—	Red, yellow, and green callus, roots
					Root	30	—	Callus
					Shoot	—	90	Direct regeneration after 30 days

0.5	1.0	—	—	—	Leaves	70	—	Yellow and red callus
					Stem	80	—	Yellow and red callus
					Root	55	—	Callus
					Shoot	—	100	Multiple shoots and roots

0.5	1.5	—	—	—	Leaves	85	—	White callus
					Stem	95	—	Orange and red callus
					Root	65	—	Callus and roots
					Shoot	—	100	Multiple shoots and roots

0.5	2.0	—	—	—	Leaves	100	—	White, red, green, and black callus
					Stem	100	—	Yellow and red callus, roots
					Root	75	—	Roots
					Shoot	—	90	Direct regeneration after 30 days

1.0	0.5	—	—	—	Leaves	35	—	White callus and roots
					Stem	55	—	Roots
					Root	5	—	Roots
					Shoot	—	100	Direct regeneration after 35 days

1.0	1.0	—	—	—	Leaves	—	—	—
					Stem	—	—	—
					Root	—	—	—
					Shoot	—	—	—

1.0	1.5	—	—	—	Leaves	65	—	White and black callus, roots
					Stem	80	—	Callus and roots
					Root	30	—	Roots
					Shoot	—	50	Multiple shoots and roots

1.0	2.0	—	—	—	Leaves	20	—	Callus
					Stem	45	—	Yellow and black callus
					Root	35	—	Roots
					Shoot	—	95	Multiple shoots and roots

1.5	0.5	—	—	—	Leaves	15	—	White callus and roots
					Stem	45	—	Roots
					Root	10	—	Roots
					Shoot	—	90	Multiple shoots and roots

1.5	1.0	—	—	—	Leaves	25	—	Roots
					Stem	60	—	Roots
					Root	10	—	Roots
					Shoot	—	100	Multiple shoots and roots

1.5	1.5	—	—	—	Leaves	45	—	Callus and roots
					Stem	85	—	Callus and roots
					Root	70	—	Roots
					Shoot	—	95	Direct regeneration after 35 days

1.5	2.0	—	—	—	Leaves	80	—	Callus and roots
					Stem	100	—	Callus and roots
					Root	70	—	Roots
					Shoot	—	100	Direct regeneration after 35 days

2.0	0.5	—	—	—	Leaves	5	—	Roots
					Stem	20	—	Roots
					Root	20	—	Roots
					Shoot	—	90	Direct regeneration after 20 days

2.0	1.0	—	—	—	Leaves	10	—	Roots
					Stem	20	—	Roots
					Root	25	—	Roots
					Shoot	—	100	Multiple shoots

2.0	1.5	—	—	—	Leaves	65	—	Callus and roots
					Stem	95	—	Orange callus and roots
					Root	75	—	Roots
					Shoot	—	100	Direct regeneration after 20 days

2.0	2.0	—	—	—	Leaves	65	—	Callus
					Stem	70	—	Yellow callus and roots
					Root	—	—	—
					Shoot	—	100	Multiple shoots

—	—	1.0	—	—	Leaves	20	—	Necrosis
					Stem	45	—	White callus and roots
					Root	15	—	Roots
					Shoot	—	100	Direct regeneration after 35 days

—	—	—	1.0	—	Leaves	5	—	Necrosis
					Stem	10	—	Roots
					Root	10	—	Roots
					Shoot	—	100	Direct regeneration after 35 days

—	—	—	—	1.0	Leaves	10	—	Necrosis
					Stem	15	—	Roots
					Root	10	—	Roots
					Shoot	—	100	Direct regeneration after 35 days

**Table 2:** The Mitotic Index and mean chromosome numbers in meristem cells of intact *Celosia cristata* plants and regenerants.

C*elosia cristata *		Mitotic Index (%)	Chromosome number (mean)
*In vivo*	Plant	39.03 ± 4.67	33.23

*In vitro*	PLANTLET		
	Normal	45.64 ± 3.16	36.05
	Abnormal	43.65 ± 2.75	36.98
	FLOWERING	46.35 ± 0.99	42.07
	NON-FLOWERING	42.27 ± 2.86	38.24

**Table 3:** The mean nuclear and cell areas and their ratios in root meristem cells of *in vivo* and *in vitro* grown *Celosia cristata* plants.

* Celosia cristata*	Mean (*μ*m^2^)		
	Nucleus (N)	Cell (C)	Ratio (N/C)
*In vivo*	49.06 ± 2.16	252.48 ± 25.88	0.19 ± 0.01
*In vitro* (normal)	84.26 ± 3.07	228.13 ± 19.25	0.37 ± 0.01
*In vitro* (abnormal)	71.24 ± 3.56	228.37 ± 13.69	0.33 ± 0.02
